# Childhood cancer incidence around nuclear installations in Great Britain, 1995–2016

**DOI:** 10.1093/ije/dyaf107

**Published:** 2025-07-16

**Authors:** Bethan Davies, Frédéric B Piel, Aina Roca-Barceló, Anna Freni Sterrantino, Hima Iyathooray Daby, Marta Blangiardo, Daniela Fecht, Frank de Vocht, Paul Elliott, Mireille B Toledano

**Affiliations:** UK Small Area Health Statistics Unit (SAHSU), Department of Epidemiology and Biostatistics, School of Public Health, Imperial College London, White City Campus, London, United Kingdom; NIHR Health Protection Research Unit in Chemical and Radiation Threats and Hazards, Imperial College London, White City Campus, London, United Kingdom; MRC Centre for Environment and Health, School of Public Health, Imperial College London, White City Campus, London, United Kingdom; UK Small Area Health Statistics Unit (SAHSU), Department of Epidemiology and Biostatistics, School of Public Health, Imperial College London, White City Campus, London, United Kingdom; NIHR Health Protection Research Unit in Chemical and Radiation Threats and Hazards, Imperial College London, White City Campus, London, United Kingdom; MRC Centre for Environment and Health, School of Public Health, Imperial College London, White City Campus, London, United Kingdom; NIHR Health Protection Research Unit in Chemical and Radiation Threats and Hazards, Imperial College London, White City Campus, London, United Kingdom; MRC Centre for Environment and Health, School of Public Health, Imperial College London, White City Campus, London, United Kingdom; The Alan Turing Institute, London, United Kingdom; UK Small Area Health Statistics Unit (SAHSU), Department of Epidemiology and Biostatistics, School of Public Health, Imperial College London, White City Campus, London, United Kingdom; NIHR Health Protection Research Unit in Chemical and Radiation Threats and Hazards, Imperial College London, White City Campus, London, United Kingdom; MRC Centre for Environment and Health, School of Public Health, Imperial College London, White City Campus, London, United Kingdom; UK Small Area Health Statistics Unit (SAHSU), Department of Epidemiology and Biostatistics, School of Public Health, Imperial College London, White City Campus, London, United Kingdom; NIHR Health Protection Research Unit in Chemical and Radiation Threats and Hazards, Imperial College London, White City Campus, London, United Kingdom; MRC Centre for Environment and Health, School of Public Health, Imperial College London, White City Campus, London, United Kingdom; Population Health Sciences, Bristol Medical School, University of Bristol, Bristol, United Kingdom; NIHR Applied Research Collaboration West (NIHR ARC West), Bristol, United Kingdom; UK Small Area Health Statistics Unit (SAHSU), Department of Epidemiology and Biostatistics, School of Public Health, Imperial College London, White City Campus, London, United Kingdom; NIHR Health Protection Research Unit in Chemical and Radiation Threats and Hazards, Imperial College London, White City Campus, London, United Kingdom; MRC Centre for Environment and Health, School of Public Health, Imperial College London, White City Campus, London, United Kingdom; NIHR Health Protection Research Unit in Chemical and Radiation Threats and Hazards, Imperial College London, White City Campus, London, United Kingdom; MRC Centre for Environment and Health, School of Public Health, Imperial College London, White City Campus, London, United Kingdom

**Keywords:** paediatric cancer, nuclear installations, epidemiology

## Abstract

**Background:**

Concerns remain about the potential harmful health impact of nuclear installations. Historical clusters of leukaemia and non-Hodgkin’s lymphoma (LNHL) in children living near Sellafield and Dounreay installations in Great Britain remain aetiologically unexplained, and the sites remain under surveillance. We assess the risk of LNHL, central nervous system (CNS) and all solid tumours in children aged 0–14 years living within 25 km of nuclear installations in Great Britain, between 1995 and 2016.

**Methods:**

We used a Poisson regression model to estimate the expected number of cases of each cancer type at the community-level in the study population, we present standardized incidence ratios compared to the national population. We used a hierarchical Poisson regression model to estimate the adjusted incidence rate ratios for each cancer type by distance between the community of residence and nearest nuclear installation.

**Results:**

We found no evidence of elevated incidence of LNHL, CNS, or all solid tumours in children resident in communities in proximity to nuclear sites. Within the 25-km zone, there was no evidence of an increased risk of childhood cancer in communities closer to installations.

**Conclusion:**

In post-1994 data, there was no evidence of an increased risk of childhood cancers in communities within 25 km of nuclear installations in Great Britain. Previously raised risks are no longer evident.

Key Messages

- We investigated whether there was an increased risk of childhood cancers during 1995–2016 in communities in proximity to nuclear installations in Great Britain.
- We did not find evidence for an increased risk of childhood cancers in communities near nuclear installations when compared with the national population. For children living within 25 km of an installation, we did not find evidence to suggest that the risk of cancer is higher in communities closer to the nuclear installations compared with the most distant communities.
- This study provides valuable new data that the excess risks of childhood leukaemia that have been observed historically in communities close to some nuclear installations in Great Britain are no longer present. The UK Committee on the Medical Aspects of Radiation in the Environment recommends that the incidence of childhood cancers in the vicinity of the Sellafield and Dounreay nuclear installations remains under surveillance and periodic review.

## Background

There are longstanding concerns about possible excesses of cancers in children living near nuclear installations. In 1983, a cluster of cases of leukaemia in children living in Seascale (Cumbria, England) close to the Sellafield nuclear reprocessing plant was reported by a Yorkshire Television programme [1, 2]. An Independent Advisory Group [3] confirmed this cluster, and the UK government established the Committee on Medical Aspects of Radiation in the Environment (COMARE) to advise on the health effects of radiation. Over the intervening 40 years, there has been extensive investigation of the associations between the nuclear industry and childhood cancer in a wide range of settings [4–12].

These studies identified increased risks of leukaemia and non-Hodgkin’s lymphoma (LNHL) in children and young adults living near Sellafield and Dounreay (Caithness, Scotland) nuclear installations before 1990 [11–13]; increased risk of leukaemia in children under 5 years old living near Krümmel (Hamburg, Germany) nuclear power plant (NPP) between 1990 and 2003 [14, 15]; and recently, increased risk of leukaemia in children living within 5 km of the Mol-Dessel (Belgium) nuclear installation between 2002 and 2016 [16, 17].

Studies in Great Britain covering 1991–2006 reported that observed excess risks of LNHL around Sellafield and Dounreay were no longer present [18]. But an evidence review by Multidisciplinary European Low Dose Initiative (MELODI) advised that post-2008 studies demonstrate non-statistically significant elevated risk of leukaemia in children aged 0–4 years living within 5 km of NPPs [10].

National small area epidemiological studies, including in Belgium, Germany, and Great Britain, have not found consistent evidence of a general increase in the risk of childhood cancer around nuclear installations [10, 18, 19]. After extensive investigations, COMARE and the German Commission on Radiological Protection independently concluded that radiation exposure from discharges from nuclear installations (Sellafield, Dounrey, and Krümmel) would have been far too small to explain the observed excess cancer cases among nearby residents [20, 21]. But these clusters of cases of LNHL were unlikely to have occurred by chance and they remain aetiologically unexplained [22], which raises concerns about health risks associated with living close to nuclear installations.

Nuclear energy is considered integral to decarbonizing energy production and meeting net zero commitments in Great Britain and internationally [23]. The Intergovernmental Panel on Climate Change recognize the contribution nuclear power could make to reducing greenhouse gases but safety concerns and associated adverse public opinion remain barriers to implementation [24]. The British government is considering increasing nuclear energy from 15% to 25% of the nation’s electricity by 2050, which would require up to eight new reactors. This has revived debates about potential health risks [25].

COMARE recommends that the Sellafield and Dounreay sites remain under surveillance and periodic review [11, 20, 22] and commissioned the UK Small Area Health Statistics Unit at Imperial to provide an independent assessment of the rates of childhood LNHL, central nervous system (CNS) and all solid tumours in the vicinity of 28 nuclear installations in Great Britain (including Sellafield and Dounreay) between 1995 and 2016. We present the results of that analysis in comparison to rates in the general population.

## Methods

### Data

We stratified the main analysis by four groups of NPPs and installations (Table 1). Group I contained 13 installations categorized as NPP by COMARE 10 and 14 reports [18, 26]. Group II comprised 13 heterogeneous nuclear installations, previously classified as ‘other’, non-NPP nuclear installations by the COMARE 10 report [18]. We diverged from the classification used by COMARE 10 by considering Dounreay (Group III) and Sellafield (Group IV) separately (not within ‘other’ category) as these were the original hypothesis-generating sites.

**Table 1.** Nuclear installations in Great Britain included in this study, grouped with reference to COMARE reports [18, 26]

Group I	Group II	Group II “major” subgroup	Group III	Group IV
Berkeley	Aldermaston	Aldermaston	Dounreay	Sellafield
Bradwell	Amersham	Amersham		
Chapelcross	Burghfield	Capenhurst		
Dungeness	Capenhurst	Harwell		
Hartlepool	Cardiff	Springfields		
Heysham	Chatham	Winfrith		
Hinkley Point	Devonport			
Hunterston	Faslane (Clyde Naval Base)			
Oldbury	Harwell			
Sizewell	Holy Loch (Loch Goil)			
Torness	Rosyth			
Trawsfynydd	Springfields			
Wylfa	Winfrith			

We defined the location of a nuclear installation as its geometric centroid based on its perimeter boundary delimited using GoogleEarth^®^ [27]. To update the most recent work by COMARE, the study period was 01 January 1995 to 31 December 2016 (latest available data). We defined ‘communities’ using 2011 Middle layer Super Output Areas (MSOA) in England and Wales (*n *= 7201; all-ages population 5000–15 000) [28] and 2011 Intermediate Zones (IZ) in Scotland (*n *= 1279; population 2500 to 6000) [29]. We obtained each community’s population-weighted centroid using the spatial location of its constituent postcodes weighted by population headcounts from the Office for National Statistics (ONS). The study area definition is consistent with previous investigations [18, 30], communities with a population-weighted centroid within 25 km of at least one nuclear installation.

We obtained person-years at risk by community by summing the annual age-specific population denominators (from ONS mid-year population estimates) across the study period.

We used government office region (*n *= 11; Scotland, Wales, and England’s nine regions) [31], community-level material deprivation, rural/urban status, and population density as covariates (see Supplementary Methods S1). Data from 2011 were used because we found high correlation between Carstairs scores at the available time points (1991, 2001, 2011; Supplementary Methods S1, Supplementary Fig. S1).

We obtained national incident cases of cancer diagnosed between 1995 and 2016 in children under 15 years of age who were resident in Great Britain from NHS England (formerly Public Health England), Welsh Cancer Intelligence and Surveillance Unit and Health Protection Scotland. We used the International Classification of Childhood Cancer 3rd edition (ICCC3) to identify LNHL (ICCC3 Ia, b, c, e, and IIb), CNS tumours (ICCC3 IIIa–f) and all solid tumours (any cancer diagnosis except ICCC3 I and II) (Supplementary Table S1). We spatially assigned incident case records to communities using 2011 geographies based on the child’s residential postcode at diagnosis and excluded cases with missing postcodes (*n *= 35) (Supplementary Methods S2).

### Statistical analysis

We use Pearsons’s chi-squared tests or *t*-tests to compare the distribution of covariates in communities in the study area to communities in Great Britain. To determine whether the occurrence of LNHL, CNS, and/or solid tumours was higher than would be expected for a population of such characteristics, we estimated the standardized incidence ratio (SIR) over the study period, defined as the ratio of observed cases to expected cases. We computed expected cases [with confidence intervals (CIs)] for each community in the study area using a Poisson regression model adjusted for Carstairs index quintile, region, and mid-year population estimate, as described in the 14th Report from COMARE, adapted from Bithell *et al.* (Supplementary Methods S3 and S4) [4, 26, 32].

To explore whether the risk of childhood LNHL, CNS, and/or all solid tumours decreased with increasing distance from a nuclear installation, we used hierarchical Poisson regression models to estimate the adjusted incidence rate ratios (aIRR) by distance between the community population-weighted centroid and the geometric centroid of the nearest nuclear installation for the communities within 25 km of a nuclear installation, accounting for rural/urban classification and population density (Supplementary Methods S5). We modelled distance as (i) a continuous variable; (ii) quartiles, with each category containing 25% of communities in the study population (distance measured to community population-weighted centroid) (Q1: <11.5 km; Q2: 11.5–16.7 km; Q3: 16.8–20.6 km; Q4: 20.7–25 km); and (iii) categories of distance, with the study area divided into 5-km distance bands. We included a random intercept for each nuclear installation to allow for differences in baseline rates as well as a community-level random effect to account for over-dispersion in the data. To comply with governance requirements and prevent secondary disclosure, we suppress observed counts ≤7 and accompanying expected counts and 95% CIs for the SIRs or aIRRs.

### Sensitivity analyses

For the complete dataset (*n *= 23), we undertook sensitivity analysis by age group (0–4, 5–9, 10–14 years), sex, and age and sex, and within the “other” category (Group II) we defined a subset of six “major” installations that undertook significant nuclear operations (compared to the rest of the group) based on Bithell *et al.* 1994 [18, 26, 32] (Table 1).

All statistical analyses were conducted in Stata 13.1 [33].

## Results

**Figure 1.**
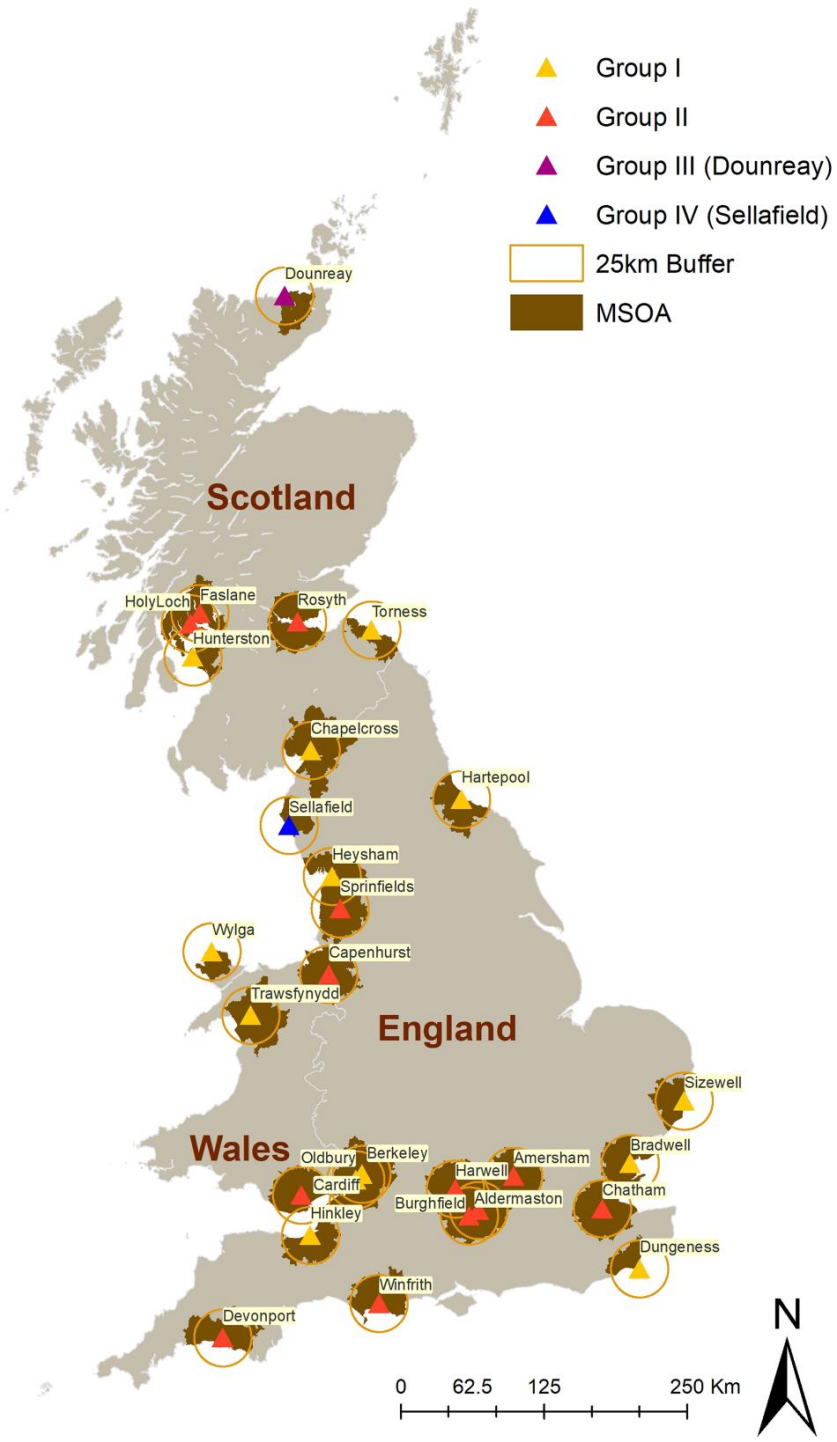
Map of nuclear installations regulated by Office for Nuclear Regulation (Group I in yellow; Group II in orange; Group III in purple, and Group IV in blue, see Table 1). The 25-km buffer around an installation’s geometric centroid is shown with the solid line, and MSOAs/IZs with a population-weighted centroid within at least one of these buffer zones are shaded.

The study area contained 23.9% (*n *= 2030/8480) of communities in Great Britain (Table 2, Fig. 1). During the study period (1995–2016), 14 791 incident cases of LNHL, 8303 cases of CNS cancer, and 27 409 cases of all solid tumours were diagnosed in children aged 0–14 years resident in Great Britain.

**Table 2.** Comparison of the demographic and socio-economic characteristics of total annual populations (i.e. person-years at risk allowing for births, migration, and deaths) at ages 0–14 years across the study period (1995–2016), for those living within the study area (within 25 km of a nuclear installation) and in Great Britain

	Study area			Great Britain		
	Population		Number of communities	Population		Number of communities
Characteristic	Person-years at risk	%		Person-years at risk	%	
Sex						
Male	28 728 995	51.2	–	121 201 253	51.2	–
Female	27 339 053	48.8	–	115 541 999	48.8	–
Age (years)						
0–4	18 505 978	33.0	–	78 713 976	33.3	–
5–9	18 674 291	33.3	–	78 811 992	33.3	–
10–14	18 887 779	33.7	–	79 190 284	33.3	–
Carstairs Index^a^						
Quintile 1	12 618 790	22.5	469	44 721 643	18.9	1697
Quintile 2	9 451 593	16.9	365	44 053 850	18.6	1696
Quintile 3	11 308 388	20.2	427	44 398 030	18.8	1695
Quintile 4	10 986 427	19.6	404	46 465 772	19.6	1696
Quintile 5	11 702 850	20.9	365	57 076 957	24.1	1696
Urban/rural						
Rural	8 580 232	15.3	331	39 343 649	16.6	1558
Urban	47 487 816	84.7	1699	197 372 603	83.4	6922
Population density						
Mean (SD)	2594 (2296)			2844 (2902)		
Total	56 068 048		2030	236 719 252		8480

a Quintile 1, least deprived; Quintile 5, most deprived. SD: standard deviation.

Compared to the under-15-years population of Great Britain, we found no evidence of elevated incidence of LNHL, CNS, or all solid tumours in the communities living in proximity to the Sellafield or Dounreay nuclear sites (CIs are wide due to small numbers) or in proximity to the group of NPPs (Group I) or the group of other nuclear installations (Group II or subset of Group II major installations) (Table 3).

**Table 3.** Observed (Obs.) and expected (Exp.) cases and standardized incidence ratio (SIR) with 95% CI for LNHL, CNS tumours, and all solid tumours for nuclear power plants (Group I), other nuclear installations (Group II), the subset of six major installations in Group II (Group II major), Dounreay (Group III), Sellafield (Group IV), 1995–2016

Group	LNHL			CNS			Solid		
	Obs.	Exp.	SIR (95% CI)	Obs.	Exp.	SIR (95% CI)	Obs.	Exp.	SIR (95% CI)
Group I	625	638.4	0.979 (0.904–1.059)	379	384.7	0.985 (0.888–1.089)	1247	1270.3	0.982 (0.928–1.038)
Group II	2166	2189.0	0.989 (0.948–1.032)	1217	1218.6	0.999 (0.943–1.056)	4016	4144.4	0.969 (0.939–0.999)
Group II (major)	1194	1185.2	1.007 (0.951–1.066)	639	639.5	0.999 (0.923–1.080)	2073	2146.2	0.966 (0.925–1.008)
Group III	^a^	^a^	0.401 (^b^–^b^)	^a^	^a^	0.752 (^b^–^b^)	^a^	^a^	1.125 (^b^–^b^)
Group IV	^b^	^b^	0.786 (^b^–^b^)	^b^	^b^	1.123 (^b^–^b^)	^b^	^b^	1.125 (^b^–^b^)

a Counts <7 suppressed.

b Removed to prevent secondary disclosure, all suppressed confidence intervals are statistically non-significant.

**Figure 2.**
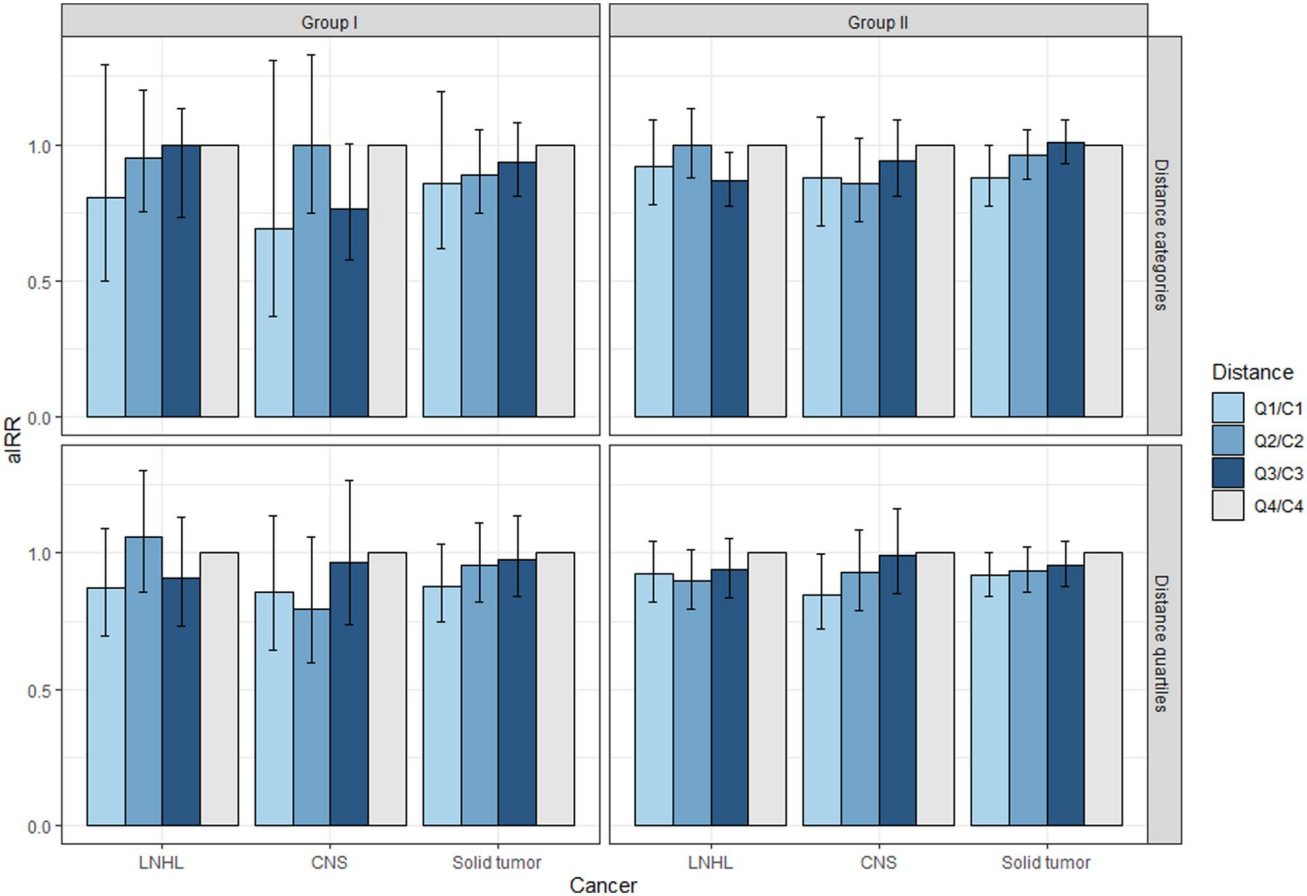
aIRR with 95% confidence intervals by categorical distance between the community population-weighted centroid and the geometric centroid of the nearest nuclear installation by cancer type for Group I and Group II in Great Britain (1995–2016). Data for Groups III and IV are not presented. The model was adjusted for age, sex, rural/urban classification, population density, deprivation (Carstairs), and region. The distance categories are as follows: (i) top panels: distance bands (5-, 10-, and 15-km cut-offs); (ii) bottom panels: 25% of communities (11.5-, 16.7-, and 20.6-km cut-offs).

Analysis by distance was not undertaken for Sellafield or Dounreay sites due to low case counts. We found no evidence of an increased risk of LNHL, CNS, or all solid tumours in communities closer to nuclear installations compared to the most distant group for Group I and Group II installations (Fig. 2, Supplementary Table S2). For Group II installations, communities 10.0–14.9 km away had a lower risk of LNHL tumours (aIRR 0.87, 95% CI 0.77–0.97), and communities <5 km away had a lower risk of all solid tumours (aIRR 0.88, 0.78–1.00) compared with communities over 15 km from the installations. In addition, 25% of communities living closest to Group II installations had a lower risk of CNS tumours (aIRR 0.85, 0.72–1.00) compared with the most distant 25% of communities.

Sensitivity analyses were consistent with the results of the main analysis (Supplementary Tables S3–S5) and only identified a single increase in incidence between the study and control areas, for LNHL in boys aged 5–9 years living in proximity to Group II nuclear installations (SIR 1.11, 1.00–1.23) (Supplementary Table S5). When limited to LNHL in boys aged 5–9 years living in proximity to the six major installations in Group II, the lower limit of the confidence interval was below 1.000 (SIR 1.14, 0.99–1.30). We further observed a decrease in all solid tumours in children aged 5–9 years living in proximity to Group I installations (SIR 0.88, 0.78–0.99) (Supplementary Table S3).

## Discussion

The excess risks of childhood LNHL that have been observed historically in communities close to Sellafield and Dounreay nuclear installations in Great Britain are no longer present. We found no evidence to suggest that children living close to nuclear installations in Great Britain between 1995 and 2016 had a higher risk of LNHL, CNS tumours, or all solid tumours compared with the general population. For children living within 25 km of an installation, we found no evidence to suggest that the risk of cancer increases with proximity to an installation.

### Strengths and limitations

We used similar methods and definitions to previous studies to undertake an updated national analysis of childhood cancer incidence near nuclear installations over a 22-year period in Great Britain [4, 20, 30]. The study was designed to generate policy-relevant findings and address the recommendations of COMARE’s 14th and 17th reports [20, 26]. We used outcome data from the UK national cancer registries which are near-complete and valid sources of cancer incidence [34].

We used straight-line distance from the population-weighted centroid of the community of residence at the time of diagnosis to the nearest nuclear installation to assign children diagnosed with cancer to either the study area or the general population. The main limitations with this approach are that we use residential location at diagnosis as a proxy for residential location at time of exposure; and exposure is assigned through distance to installations.

Cancer registry data in Great Britain do not allow retrospective (time-varying) address profiles to be constructed. Such data would be a major advance for environmental epidemiology studies particularly for diseases like cancer where there can be a long latent period between exposure and diagnosis. Using residential information from the date of diagnosis to assign exposure potentially misclassifies the exposure status of children who moved house between exposure and their diagnosis. We consider that this misclassification is likely to impact equally on children moving in either direction between study and control areas (i.e. a non-differential misclassification) which would act to bias effect estimates towards the null and could lead to false negative findings. Therefore, to improve the accuracy of future studies, generating time-varying residential location history in administrative data should be prioritized in health data strategy [35].

Our ecological approach based on residential postcode and distance to the closest installation has limitations as a proxy for potential exposure to ionizing radiation due to the complex exposure pathways between an installation and a residence. The Geocap study used community-level estimated bone marrow doses based on gaseous radioactive discharges from installations and found that while this changed the categorization of communities compared to a distance-based approach, both exposure methods produced the same findings of no association between acute leukaemia incidence and exposure to an installation [5]. Area-level exposures may misclassify participants, but we used small geographical units and population-weighted centroids to minimize this risk. Furthermore, we were unable to account for time spent away from the home but given the impossibility of collecting individual-level data by, for example, individual monitoring of radiation exposures, this distance-based approach is the accepted standard for population-based studies. We were also only able to adjust for area-level confounding; nonetheless, our study strengthens inferences from the wider literature that includes case–control studies of children living in proximity to nuclear installations where individual-level confounders were considered. Furthermore, our analysis assumes that the potential for impacts on health from radionuclides released from an installation was present and constant from the date of opening to the end of the study, given the long half-life of radionuclides and uncertainty about exposure pathways [26].

Statistical disclosure control requirements from data providers prevent presentation of complete count data from our analysis due to small numbers of cases. Therefore, we cannot provide the level of transparency that has previously been possible in this field [20].

### Study findings in context

Investigation of childhood cancer and nuclear installations in Great Britain is extensively documented. The cluster of cases of leukaemia in young people living in Seascale (1955–92) was confirmed by an Independent Group after being reported by a television documentary investigating potential occupational risks at Sellafield [3, 11]. The cluster of childhood leukaemia cases in Thurso was found when Dounreay was studied as the other nuclear reprocessing facility in the UK [13]. Finally, clusters of cases of leukaemia and lymphatic cancer around the Atomic Weapons Research Establishment (Aldermaston) and nearby Royal Ordinance Factory (Burghfield) were confirmed following concerns raised by Dr Carol Barton (consultant haematologist) and a 1985 television documentary [36]. National studies did not find evidence of an increased risk of LNHL more generally around nuclear installations in England and Wales between 1966 and 1987 [32], in Scotland between 1968 and 1993 [37], nor in Great Britain between 1969 and 1993 [18].

Our work is in keeping with previous publications from COMARE and others that have shown that the clusters of cancer identified in proximity to Sellafield and Dounreay between 1955 and 1991 are no longer present after 1991 [20, 22]. We undertook extensive sensitivity analysis that identified one excess risk between residential proximity and cancer incidence; an increase in risk of LNHL for boys aged 5–9 years living in proximity to the heterogenous group of non-NPP installations. The overall pattern of our results does not show evidence of an increased risk related to proximity to nuclear installations. Therefore, in the absence of any other excess risks, this is likely a chance finding.

The historical clusters of cases in Seascale and Thurso remain aetiologically unexplained. Extensive radiological investigations indicated that radioactive discharges into the environment were probably insufficient to cause the observed excess cases of cancer in these settings: at Sellafield, the site with the largest discharges (estimated to be 200 000 times higher than those from Aldermaston and Burghfield) the discharges would have needed to be 200 times greater “to cause observed excess of cases of LNHL” based on the assumed dose-response relationship [11, 18, 36].

A range of other aetiologies have been explored. Paternal pre-conceptional irradiation [38] was excluded after case–control and cohort studies [39–42]. Kinlen proposed population mixing [43–45], whereby the in-migration of workers and their families—needed for the construction/operation of installations—introduces a currently unidentified leukaemogenic virus to the susceptible local population; it is hypothesized that this initiates a subclinical epidemic and triggers leukaemic transformation in a proportion of children, possibly due to delayed exposure to the infection [46]. The excess incidence and mortality from LNHL in young people in Great Britain were found to be similar between sites with nuclear installations and those earmarked for development where installations were not built, which suggests that these sites may share unknown risk factors unrelated to nuclear operations [7, 32].

Clusters of cases of childhood leukaemia and lymphoma have been reported in a range of global settings [47, 48], often not associated with nuclear installations. The EUROCLUS project exploring spatial clustering of childhood leukaemia between 1980 and 1989 in 17 European countries found no evidence of clustering associated with environmental hazards (including nuclear installations) but potential patterning associated with population density and demographic factors which lends some support to an infectious aetiology hypothesis for some clusters [49, 50]. However, a national analysis of the spatiotemporal distribution of childhood cancers in Great Britain by COMARE suggests that while there is a tendency for cases of childhood leukaemia to cluster, this clustering was not explained by community-level socio-demographic factors [22].

The consensus is that there were unidentified factors in common in the communities of Seascale and Thurso that may have contributed to the shared historical risk of LNHL, but which no longer seem to be present.

## Conclusion

In this updated surveillance of communities living in proximity to nuclear installations in Great Britain, we did not find evidence for an increased risk of childhood LNHL, CNS tumours, or all solid tumours compared with the national population. While in the words of COMARE, ‘no study can show that there is no risk’ [26], we argue that this detailed study provides valuable new data that do not show persistence of health effects around nuclear installations over the longer term. Nonetheless, ongoing surveillance of these sites seems warranted. Our results are timely given renewed interest in nuclear power in the context of an energy crisis, geopolitical tensions, and zero-emission targets.

## Ethics approval

The UK Small Area Health Statistics Unit (SAHSU) holds approvals from the London-South East Research Ethics Committee (22/LO/0256) and support from the Health Research Authority Confidentiality Advisory Group (20/CAG/0028) under Regulation 5 of the Health Service (Control of Patient Information) Regulations 2002 to process confidential patient information without consent.

## Supplementary Material

dyaf107_Supplementary_Data

## Data Availability

The data underlying this article was provided NHS England, Welsh Cancer Intelligence and Surveillance Unit and Public Health Scotland. The data cannot be shared publicly by the authors because SAHSU does not have permission to supply data to third parties. We advise that the data providers are contacted directly.
