# Supplementary material for: Childhood cancer incidence around nuclear installations in Great Britain, 1995–2016
Source: Int J Epidemiol. 2025 Jul 16;54(4):dyaf107. doi: 10.1093/ije/dyaf107 (PMC12263190; doi:10.1093/ije/dyaf107)
Supplement: dyaf107_Supplementary_Data [file dyaf107_supplementary_data.docx]

**Childhood cancer incidence around nuclear installations in Great Britain, 1995-2016**

Bethan Davies, Frédéric B. Piel, Aina Roca-Barceló, Anna Freni Sterrantino, Hima Iyathooray Daby, Marta Blangiardo, Daniela Fecht, Frank De Vocht, Paul Elliott, Mireille B. Toledano

**SUPPLEMENTARY FILE**

**Methods S1. Description of confounder data**

To measure material deprivation for each community, we computed Carstairs Index, a composite, area-level indicator of material deprivation, in a consistent manner for England, Scotland and Wales based on 2011 ONS Census variables on low social class, lack of car ownership, household overcrowding and male unemployment, which we standardized across communities in Great Britain and categorised into quintiles. We used the ONS rural/urban classification, available at Census Output Areas (COA) level (around 300 people), that defines built-up areas with a population of >10 000 as urban. We aggregated the COA classification to community level by categorising communities as urban if more than 50% of the constituent COAs were urban. We calculated population density for each community as the total population per km^2^, using total population counts from the 2011 Census.

The study period, 1995-2016, is covered by three different UK censuses (1991, 2001 and 2011). For each of the studied covariates, we collected the information for all census years and harmonized the values to match the Middle layer Super Output Area (MSOA) boundaries of the 2011 census. Once all information was harmonized at the same geographical level, we explored the variation in the variables over time using correlations across the different census years (or Chi squared for categorical variables). Supplementary Figure 1 shows the correlation between the Carstairs scores (material deprivation) across different census years (correlation coefficients: >0.9). Overall, for Carstairs and population density, the correlation between census years was high and there was no evidence of a ~~d~~ifference in the distribution of rural/urban categorisation so we decided to use information from the 2011 census in this study. This was a compromise between potential bias from temporal trends not captured by this approach and loss of statistical power from stratified analyses (by census period).

**Method S2: Missing postcodes**

There were 35 individuals in England and Wales for whom the postcode recorded in the cancer registry could not be matched to a specific postcode area in our database. Although we could not explore specific reasons for each case, this is likely due to errors in data entry in the postcode recorded for these individuals. They were therefore excluded both from the numerators and denominators in our analyses. We acknowledge that this approach may have led to a potential bias in estimating the risks, however this is likely to be small.

**Method S3: Expected count**

The expected number of childhood cancer cases was calculated using the approach described in COMARE’s 14^th^ Report and adapted from Bithell *et al.*^[[1]](#footnote-2),^^[[2]](#footnote-3)^ so that:

$e_{i}=$*N_i_ exp(µ + α_k(i)_ + β_f(i)_ + γ_t(i)_)*

Where *N_i_* is the mid-year under-15 population estimate, *k(i)* is the Carstairs Index quintile of the i-th community, *f(i)* is an indicator of the region the i-th community belongs to and *t(i)* is the i-th community rural/urban classification. Note that all the covariates are included in the model as categorical variables, so the first category is set as the reference (*α_1_=β_1_=γ_1_*=0).

**Method S4: Confidence Intervals for Expected Counts**

We estimated 95% Confidence Intervals (CI) using Equations 1a and 1b as an approximation,^[[3]](#footnote-4)^ where *Obs.* are observed counts, *Exp.* are expected counts and z is standardized normal deviate corresponding to α/2 (1.96 for 95% CI).

*SIR_Lower_ = Obs. [1 - (9 Obs.)^-1^ – z (9 Obs.)^-1/2^]^3^/Exp.* Eq. 1a

*SIR_Upper_ = (Obs. +1) [1 - 9(Obs.+1))^-1^ + z (9 (Obs.+1)) ^-1/2^]^3^/Exp.* Eq. 1b

**Methods S5: Adjusted incidence rate ratio (aIRR)**

We used hierarchical Poisson regression models to estimate the adjusted incidence rate ratios (aIRR) by distance between the community population-weighted centroid and the geometric centroid of the nearest nuclear installation, accounting for rural/urban classification and population density. We define a categorical variable *Dist*, which summarises the distance from the nuclear installation for all the communities, so that Dist=1,..,j,..,J (*J* is the furthest distance and is assumed as reference in the model). For each community i the model is the following:

$$O_{i} \sim Poisson (\lambda_{i}E_{i})$$

$$\log\left( \lambda_{i} \right)= \beta_{0}+\beta_{1Urb_{i}}+\beta_{2}{Pop\delta}_{i}+\gamma_{Dist_{i}}+u_{i}$$

where $O_{i}$is the adjusted observed childhood cancer count at community i; $e_{i}$ is the estimated childhood cancer count for community i based on the equation in S3. Then $\gamma_{Dist_{i}}$ represents the regression coefficient for the j category of distance from the nuclear installation compared to the reference; ${Urb}_{i}$ is a measure of urbanicity, ${Pop\delta}_{i}$ is the population density, $\beta_{0}$ is the intercept; $\boldsymbol{\beta}_{1} and \beta_{2}$ are regression coefficients; $u_{i}$ is a random effect.

Finally, $aIRR= \frac{\lambda_{j}e_{j}}{\lambda_{J}e_{J}}=\frac{\sum_{i\in Dist=j} \lambda_{i}e_{i}/N_{i\in Dist=j}}{\sum_{i\in Dist=j} \lambda_{i}e_{i}/N_{i\in Dist=J}}$

Confidence intervals for the aIRRs were calculated using the following formula:

$$95\% CI=exp(log(aIRR)\pm1.96\times SE)$$

$$SE = \sqrt{\frac{1}{n_{d1}}+\frac{1}{n_{d2}}+\frac{1}{n_{d3}}+\frac{1}{n_{d4}}}$$

*Where n_dj_ is the number of cases in distance band j.*

An alternative model considers the distance from the installation as a continuous variable; the specification on the $\log\left( \lambda_{i} \right)$above becomes

$$\log\left( \lambda_{i} \right)= \beta_{0}+\beta_{1}{Urb}_{i}+\beta_{2}{Pop\delta}_{i}+\gamma Dist_{i}+u_{i}$$

and $aIRR= \frac{\lambda_{j}e_{j}}{\lambda_{j-1}e_{j-1}}=\frac{\sum_{i\in Dist=j} \lambda_{i}e_{i}/N_{i\in Dist=j}}{\sum_{i\in Dist=j-1} \lambda_{i}e_{i}/N_{i\in Dist=j-1}}$

**Figure S1.** Correlation between Carstairs index scores from 1991, 2001 and 2011, using Middle layer Super Output Area (MSOA) boundaries for 2011. Pearson’s correlation coefficients are 0.9474, 0.9588 and 0.9092, respectively.

**
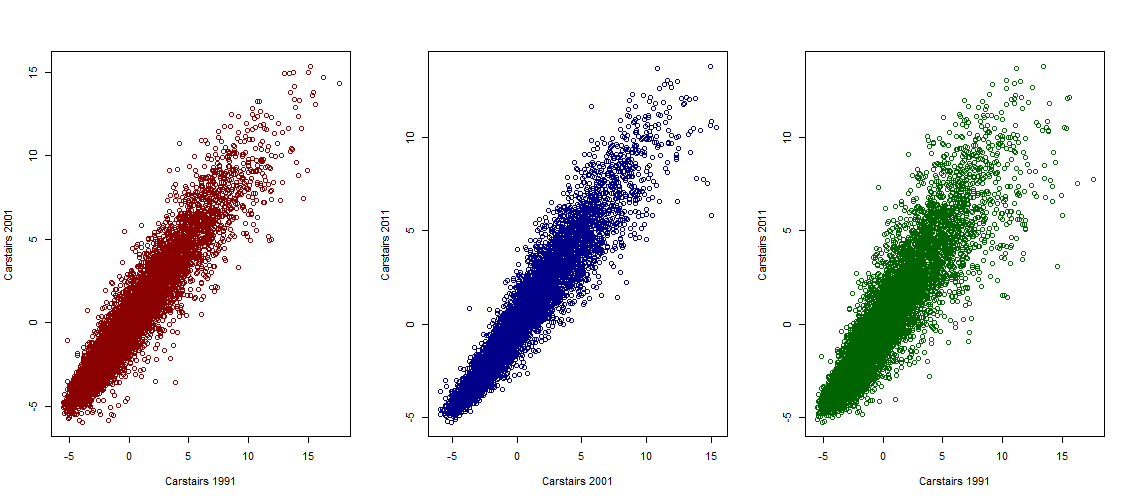
**

**Table S1**: International Childhood Cancer Classification 3rd edition (ICCC3) based on ICD-O-3 and updated for Hematopoietic codes based on WHO Classification of Tumours of Haematopoietic and Lymphoid Tissues (2008).

| Site Group | ICD-O-3 Histology (Type) | ICD-O-2/3 Site | Recode |
| --- | --- | --- | --- |
| I. Leukaemia, myeloproliferative diseases, and myelodysplastic diseases | | | |
| (a) Lymphoid leukaemia | 9820, 9823, 9826, 9827, 9831-9837, 9940, 9948 | C000-C809 | 11 |
| (b) Acute myeloid leukaemia’s | 9840, 9861, 9866, 9867, 9870-9874, 9891, 9895-9897, 9910, 9920, 9931 | C000-C809 | 12 |
| (c) Chronic myeloproliferative diseases | 9863, 9875, 9876, 9950, 9960-9964 | C000-C809 | 13 |
| (e) Unspecified and other specified leukaemia | 9800, 9801, 9805, 9860, 9930 | C000-C809 | 15 |
| II. Lymphomas and reticuloendothelial neoplasms | | | |
| (b) Non-Hodgkin lymphomas (except Burkitt lymphoma) | 9591, 9670, 9671, 9673, 9675, 9678-9680, 9684, 9689-9691, 9695, 9698-9702, 9705, 9708, 9709, 9714, 9716-9719, 9727-9729, 9731-9734, 9760-9762, 9764-9769, 9970 | C000-C809 | 22 |
| III. CNS and miscellaneous intracranial and intraspinal neoplasms | | | |
| (a) Ependymomas and choroid plexus tumour | 9383, 9390-9394 | C000-C809 | 31 |
| (b) Astrocytoma | 9380 | C723 | 32 |
|  | 9384, 9400-9411, 9420, 9421-9424, 9440-9442 | C000-C809 | 32 |
| (c) Intracranial and intraspinal embryonal tumours | 9470-9474, 9480, 9508 | C000-C809 | 33 |
|  | 9501-9504 | C700-C729 | 33 |
| (d) Other gliomas | 9380 | C700-C722, C724-C729, C751, C753 | 34 |
|  | 9381, 9382, 9430, 9444, 9450, 9451, 9460 | C000-C809 | 34 |
| (e) Other specified intracranial and intraspinal neoplasms | 8270-8281, 8300, 9350-9352, 9360-9362, 9412, 9413, 9492, 9493, 9505-9507, 9530-9539, 9582 | C000-C809 | 35 |
| (f) Unspecified intracranial and intraspinal neoplasms | 8000-8005 | C700-C729, C751-C753 | 36 |

**Table S2**. Incidence rate ratios (IRR) and adjusted IRRs (adjusted for age, sex, rural-urban, population density, deprivation and region) with 95% confidence intervals for Leukaemia and Non-Hodgkin’s Lymphoma (LNHL), Central Nervous System (CNS) tumours and Solid tumours by distance between community population-weighted centroid and geometric centroid of the nearest nuclear installation, for nuclear power plants (Group I) and other nuclear facilities (Group II) in Great Britain (1995-2016). Distance modelled as a continuous variable, as quartiles each containing 25% of communities in the study area, and as categories defined as 5km distance bands.

|  |  | **Incidence Rate Ratio (95% CI)** | | | **Adjusted Incidence Rate Ratio (95% CI)** | | |
| --- | --- | --- | --- | --- | --- | --- | --- |
| **Group** | **Distance** | **LNHL** | **CNS** | **Solid** | **LNHL** | **CNS** | **Solid** |
| **I** | **Continuous (km)** | 1.000 (1.000-1.000) | 1.000 (1.000-1.000) | 1.000 (1.000-1.000) | 1.000 (1.000-1.000) | 1.000 (1.000-1.000) | 1.000 (1.000-1.000) |
|  | **Quartiles (km)** |  |  |  |  |  |  |
|  | 4 - (>20.6) | 1.000 | 1.000 | 1.000 | 1.000 | 1.000 | 1.000 |
|  | 3 - (16.8 - 20.6) | 0.910 (0.732-1.132) | 0.970 (0.741-1.270) | 0.977 (0.842-1.135) | 0.910 (0.732-1.132) | 0.967 (0.738-1.266) | 0.977 (0.842-1.135) |
|  | 2 - (11.5 - 16.7) | 1.045 (0.848-1.287) | 0.815 (0.616-1.080) | 0.964 (0.829-1.121) | 1.057 (0.856-1.305) | 0.796 (0.600-1.057) | 0.954 (0.819-1.111) |
|  | 1 - (<11.5) | 0.868 (0.693-1.086) | 0.856 (0.645-1.136) | 0.883 (0.752-1.036) | 0.870 (0.695-1.089) | 0.856 (0.645-1.136) | 0.880 (0.749-1.033) |
|  | **Categories (km)** |  |  |  |  |  |  |
|  | 4 - (>14.9) | 1.000 | 1.000 | 1.000 | 1.000 | 1.000 | 1.000 |
|  | 3 - (10.0- 14.9) | 0.991 (0.812-1.209) | 0.777 (0.591-1.022) | 0.944 (0.819-1.089) | 0.997 (0.732-1.132) | 0.762 (0.579-1.003) | 0.937 (0.811-1.082) |
|  | 2 - (5.0 - 9.9) | 0.950 (0.755-1.195) | 0.983 (0.741-1.303) | 0.890 (0.751-1.054) | 0.951 (0.755-1.198) | 0.999 (0.751-1.328) | 0.887 (0.749-1.052) |
|  | 1 - (<5) | 0.804 (0.501-1.289) | 0.713 (0.379-1.342) | 0.864 (0.624-1.196) | 0.806 (0.502-1.295) | 0.693 (0.368-1.307) | 0.860 (0.621-1.193) |
| **II** | **Continuous (km)** | 1.000 (1.000-1.000) | 1.000 (1.000-1.000) | 1.000 (1.000-1.000) | 1.000 (1.000-1.000) | 1.000 (1.000-1.000) | 1.000 (1.000-1.000) |
|  | **Quartiles (km)** |  |  |  |  |  |  |
|  | 4 - (>20.6) | 1.000 | 1.000 | 1.000 | 1.000 | 1.000 | 1.000 |
|  | 3 - (16.8 - 20.6) | 0.939 (0.834-1.056) | 0.994 (0.851-1.162) | 0.957 (0.877-1.045) | 0.939 (0.835-1.056) | 0.994 (0.851-1.162) | 0.958 (0.878-1.045) |
|  | 2 - (11.5 - 16.7) | 0.898 (0.796-1.012) | 0.932 (0.794-1.093) | 0.940 (0.860-1.026) | 0.898 (0.797-1.013) | 0.927 (0.790-1.087) | 0.935 (0.856-1.022) |
|  | 1 - (<11.5) | 0.926 (0.823-1.042) | 0.852 (0.725-1.001) | 0.922 (0.845-1.006) | 0.926 (0.823-1.043) | 0.849 (0.723-0.998) | 0.918 (0.841-1.002) |
|  | **Categories (km)** |  |  |  |  |  |  |
|  | 4 - (>14.9) | 1.000 | 1.000 | 1.000 | 1.000 | 1.000 | 1.000 |
|  | 3 - (10.0- 14.9) | 0.867 (0.774-0.971) | 0.946 (0.818-1.094) | 1.014 (0.937-1.098) | 0.867 (0.774-0.972) | 0.941 (0.813-1.089) | 1.008 (0.931-1.091) |
|  | 2 - (5.0 - 9.9) | 0.998 (0.881-1.130) | 0.806 (0.722-1.024) | 0.964 (0.879-1.057) | 0.998 (0.880-1.130) | 0.857 (0.719-1.021) | 0.959 (0.874-1.052) |
|  | 1 - (<5) | 0.922 (0.778-1.091) | 0.876 (0.698-1.099) | 0.877 (0.774-0.994) | 0.922 (0.779-1.091) | 0.878 (0.700-1.102) | 0.880 (0.777-0.998) |

**Table S3**. Observed (Obs.) and expected (Exp.) cases, and standardized incidence ratio (SIR) with 95% confidence intervals for Leukaemia and Non-Hodgkin’s Lymphoma (LNHL), Central Nervous System (CNS) tumours and Solid tumours across nuclear power plants (Group I), other nuclear facilities (Group II), Dounreay (Group III) and Sellafield (Group IV) in Great Britain (1995-2016), stratified by age.

| **Age Group** | **Installation Group** | **LNHL** | | | **CNS** | | | **Solid** | | |
| --- | --- | --- | --- | --- | --- | --- | --- | --- | --- | --- |
|  |  | **Obs.** | **Exp.** | **SIR (95% CI)** | **Obs.** | **Exp.** | **SIR (95% CI)** | **Obs.** | **Exp.** | **SIR (95% CI)** |
| **0-4 years** | I | 302 | 316.4 | 0.955 (0.850-1.069) | 148 | 144.3 | 1.026 (0.867-1.205) | 594 | 580.6 | 1.023 (0.942-1.109) |
|  | II | 1061 | 1093.8 | 0.970 (0.912-1.030) | 455 | 462.5 | 0.984 (0.896-1.079) | 1820 | 1883.9 | 0.966 (0.922-1.012) |
|  | III | * | * | 0.849 ** | * | * | 1.986 ** | * | * | 1.555 |
|  | IV | * | * | 0.842 ** | * | * | 1.047 ** | ** | * | 0.915 ** |
| **5-9 years** | I | 177 | 177.7 | 0.996 (0.855-1.154) | 116 | 133.4 | 0.870 (0.719-1.043) | 265 | 300.9 | 0.881 (0.778-0.993) |
|  | II | 634 | 601.6 | 1.054 (0.973-1.139) | 449 | 445.9 | 1.007 (0.916-1.105) | 987 | 1031.0 | 0.957 (0.899-1.019) |
|  | III | * | * | 0.000 ** | * | * | 0.000 ** | * | * | 0.000 ** |
|  | IV | * | * | 1.221 ** | * | * | 0.987 ** | * | * | 0.878 ** |
| **10-14 years** | I | 146 | 144.1 | 1.013 (0.856-1.192) | 115 | 107.0 | 1.075 (0.888-1.291) | 388 | 388.6 | 0.998 (0.901-1.103) |
|  | II | 471 | 495.8 | 0.950 (0.866-1.040) | 313 | 310.9 | 1.007 (0.898-1.125) | 1209 | 1231.9 | 0.981 (0.927-1.038) |
|  | III | * | * | 0.000 ** | * | * | 0.000 ** | * | * | 1.413 ** |
|  | IV | * | * | 0.257 ** | * | * | 1.455 ** | 14 | 8.5 | 1.650 (0.901-2.768) |

* Small number (n≤7) suppressed; ** 95% confidence intervals not presented to prevent secondary disclosure, no statistically significant results.

**Table S4**. Observed (Obs.) and expected (Exp.) cases, and standardized incidence ratio (SIR) with 95% confidence intervals for Leukaemia and Non-Hodgkin’s Lymphoma (LNHL), Central Nervous System (CNS) tumours and Solid tumours across nuclear power plants (Group I), other nuclear facilities (Group II), Dounreay (Group III) and Sellafield (Group IV) in Great Britain (1995-2016), stratified by sex.

| **Sex** | **Group** | **LNHL** | | | **CNS** | | | **Solid** | | |
| --- | --- | --- | --- | --- | --- | --- | --- | --- | --- | --- |
|  |  | **Obs.** | **Exp.** | **SIR (95% CI)** | **Obs.** | **Exp.** | **SIR (95% CI)** | **Obs.** | **Exp.** | **SIR (95% CI)** |
| Male | I | 354 | 365.2 | 0.969 (0.871-1.076) | 199 | 199.975 | 0.995 (0.862-1.143) | 654 | 642.6 | 1.018 (0.941-1.099) |
|  | II | 1250 | 1245.8 | 1.003 (0.949-1.061) | 656 | 648.05 | 1.012 (0.936-1.093) | 2012 | 2093.5 | 0.961 (0.920-1.004) |
|  | III | * | * | 0.698 ** | * | * | 1.465 ** | * | * | 1.336 ** |
|  | IV | * | * | 0.593 ** | * | * | 0.693 ** | 14 | 14 | 1.002 (0.547-1.681) |
| Female | I | 271 | 273.3 | 0.991 (0.878-1.117) | 180 | 184.727 | 0.974 (0.837-1.128) | 593 | 627.7 | 0.945 (0.870-1.024) |
|  | II | 916 | 943.1 | 0.971 (0.909-1.036) | 561 | 570.658 | 0.983 (0.903-1.068) | 2004 | 2051 | 0.977 (0.935-1.021) |
|  | III | * | * | 0.000 ** | * | * | 0.000 ** | * | * | 0.910 ** |
|  | IV | * | * | 1.023 ** | * | * | 1.629 ** | 17 | 13.6 | 1.253 (0.729-2.006) |

* Small number (n≤7) suppressed; ** 95% confidence intervals not presented to prevent secondary disclosure.

**Table S5**. Observed (Obs.) and expected (Exp.) cases, and standardized incidence ratio (SIR) with 95% confidence intervals (95% CI) for Leukaemia and Non-Hodgkin’s Lymphoma (LNHL), Central Nervous System (CNS) tumours and Solid tumours across nuclear power plants (Group I), other nuclear facilities (Group II), Dounreay (Group III) and Sellafield (Group IV) in Great Britain (1995-2016), stratified by age and sex.

| **Age Group** | **Sex** |  | **LNHL** | | | **CNS** | | | **Solid** | | |
| --- | --- | --- | --- | --- | --- | --- | --- | --- | --- | --- | --- |
|  |  | **Group** | **Obs.** | **Exp.** | **SIR (95% CI)** | **Obs.** | **Exp.** | **SIR (95% CI)** | **Obs.** | **Exp.** | **SIR (95% CI)** |
| 0-4 years | Male | I | 171 | 173.8 | 0.984 (0.842-1.143) | 69 | 69.6 | 0.991 (0.771-1.255) | 321 | 296.2 | 1.084 (0.968-1.209) |
|  |  | II | 572 | 591.2 | 0.967 (0.890-1.050) | 244 | 247.3 | 0.987 (0.867-1.119) | 922 | 982.8 | 0.938 (0.879-1.001) |
|  |  | III | * | * | 1.470 ** | * | * | 4.010 ** | * | * | 0.980 ** |
|  |  | IV | * | * | 0.262 ** | * | * | 1.188 ** | * | * | 0.936 ** |
|  | Female | I | 131 | 142.6 | 0.919 (0.768-1.090) | 79 | 74.6 | 1.059 (0.839-1.320) | 273 | 284.3 | 0.960 (0.850-1.081) |
|  |  | II | 489 | 502.5 | 0.973 (0.889-1.063) | 211 | 215.2 | 0.981 (0.853-1.122) | 898 | 900.8 | 0.997 (0.933-1.064) |
|  |  | III | * | * | 0.000 ** | * | * | 0.000 ** | * | * | 2.199 ** |
|  |  | IV | * | * | 1.519 ** | * | * | 0.836 ** | * | * | 0.893 ** |
| 5-9 years | Male | I | 99 | 106.3 | 0.931 (0.757-1.134) | 65 | 74.7 | 0.870 (0.671-1.109) | 141 | 158.5 | 0.890 (0.749-1.049) |
|  |  | II | 397 | 357.5 | 1.110 (1.004-1.225) | 254 | 244.1 | 1.041 (0.916-1.177) | 524 | 529.5 | 0.990 (0.907-1.078) |
|  |  | III | * | * | 0.000 ** | * | * | 0.000 ** | * | * | 0.000 ** |
|  |  | IV | * | * | 1.302 ** | * | * | 0.000 ** | * | * | 0.295 ** |
|  | Female | I | 78 | 71.4 | 1.092 (0.863-1.363) | 51 | 58.7 | 0.869 (0.647-1.124) | 124 | 142.5 | 0.870 (0.724-1.038) |
|  |  | II | 237 | 244.0 | 0.971 (0.852-1.103) | 195 | 201.8 | 0.967 (0.836-1.112) | 463 | 501.5 | 0.923 (0.841-1.011) |
|  |  | III | * | * | 0.000 ** | * | * | 0.000 ** | * | * | 0.000 ** |
|  |  | IV | * | * | 1.113 ** | * | * | 2.050 ** | * | * | 1.452 ** |
| 10-14 years | Male | I | 84 | 85.0 | 0.988 (0.788-1.223) | 65 | 55.4 | 1.173 (0.906-1.496) | 192 | 187.7 | 1.023 (0.883-1.178) |
|  |  | II | 281 | 298.0 | 0.943 (0.836-1.060) | 158 | 157.0 | 1.006 (0.855-1.176) | 566 | 583.1 | 0.971 (0.892-1.054) |
|  |  | III | * | * | 0.000 ** | * | * | 0.000 ** | * | * | 3.024 ** |
|  |  | IV | * | * | 0.451 ** | * | * | 0.959 ** | * | * | 1.736 ** |
|  | Female | I | 62 | 59.0 | 1.050 (0.805-1.346) | 50 | 51.5 | 0.971 (0.720-1.280) | 196 | 200.9 | 0.976 (0.844-1.122) |
|  |  | II | 190 | 197.9 | 0.960 (0.829-1.107) | 155 | 154.0 | 1.007 (0.854-1.178) | 643 | 649.1 | 0.991 (0.916-1.070) |
|  |  | III | * | * | 0.000 ** | * | * | 0.000 ** | * | * | 0.000 ** |
|  |  | IV | * | * | 0.000 ** | * | * | 1.966 ** | * | * | 1.574 ** |

* Small number (n≤7) suppressed; ** 95% confidence intervals not presented to prevent secondary disclosure.

1. Bithell, J.F., et al., Distribution of childhood leukaemias and non-Hodgkin's lymphomas near nuclear installations in England and Wales. Bmj, 1994. 309(6953): p. 501-5.

   [↑](#footnote-ref-2)
2. Committee on the Medical Aspects of Radiation in the Environment, Fourteenth report. Further consideration of the incidence of childhood leukaemia around nuclear power plants in Great Britain., H.P. Agency, Editor. 2011: Chilton [↑](#footnote-ref-3)
3. Liddell FD. Simple exact analysis of the standardised mortality ratio. *J Epidemiol Community Health* 1984; **38**: 85-8. [↑](#footnote-ref-4)
